# Clinical Value of Complex Echocardiographic Left Ventricular Hypertrophy Classification Based on Concentricity, Mass, and Volume Quantification

**DOI:** 10.3389/fcvm.2021.667984

**Published:** 2021-04-27

**Authors:** Andrea Barbieri, Alessandro Albini, Anna Maisano, Gerardo De Mitri, Giovanni Camaioni, Niccolò Bonini, Francesca Mantovani, Giuseppe Boriani

**Affiliations:** ^1^Division of Cardiology, Department of Diagnostics, Clinical and Public Health Medicine, Policlinico University Hospital of Modena, University of Modena and Reggio Emilia, Modena, Italy; ^2^Cardiology, Azienda USL-IRCCS di Reggio Emilia, Reggio Emilia, Italy

**Keywords:** left ventricular mass, left ventricular function, left ventricular volume, echocardiograghy, clinical value, prognosis

## Abstract

Echocardiography is the most validated, non-invasive and used approach to assess left ventricular hypertrophy (LVH). Alternative methods, specifically magnetic resonance imaging, provide high cost and practical challenges in large scale clinical application. To include a wide range of physiological and pathological conditions, LVH should be considered in conjunction with the LV remodeling assessment. The universally known 2-group classification of LVH only considers the estimation of LV mass and relative wall thickness (RWT) to be classifying variables. However, knowledge of the 2-group patterns provides particularly limited incremental prognostic information beyond LVH. Conversely, LV enlargement conveys independent prognostic utility beyond LV mass for incident heart failure. Therefore, a 4-group LVH subdivision based on LV mass, LV volume, and RWT has been recently suggested. This novel LVH classification is characterized by distinct differences in cardiac function, allowing clinicians to distinguish between different LV hemodynamic stress adaptations in various cardiovascular diseases. The new 4-group LVH classification has the advantage of optimizing the LVH diagnostic approach and the potential to improve the identification of maladaptive responses that warrant targeted therapy. In this review, we summarize the current knowledge on clinical value of this refinement of the LVH classification, emphasizing the role of echocardiography in applying contemporary proposed indexation methods and partition values.

## Introduction

Nowadays, the perpetual controversy between the importance of structural and functional anomalies in the failed heart appears to lack consensus (1). On the one side, the left ventricular (LV) ejection fraction (EF) calculation informs many care decisions for heart failure, on the other hand, using modern echocardiographic techniques, the quantification of LV mass and geometry is highly feasible and with a single diagnostic exam. Especially, left ventricular hypertrophy (LVH), when defined by increased ventricular mass according to the classification and partition values proposed by the American Society of Echocardiography/European Association of Cardiovascular Imaging (ASE/EACVI) (2), is a strong independent predictor of cardiovascular risk in adults undergoing assessment for any indication (3).

Importantly, in order to cover a broad variety of physiological and pathological disorders, LVH should be considered according to the classification of LV remodeling, which is the initial step of cardiac damage. The most commonly used categorization for LVH remodeling patterns is proposed by the ASE/EACVI, which uses only LV mass and relative wall thickness (RWT) as classifying variables with two known basic patterns: concentric and eccentric LVH (2). However, although patients with concentric LVH have a different clinical and biomarker phenotype compared to those with eccentric LVH (4), the knowledge of these remodeling patterns provided particularly limited incremental prognostic information beyond LVH *per se*. A recent systematic review and network meta-analysis of 22 echocardiographic publications (76.133 individuals) studied across various patient populations showed that concentric and eccentric LVH was associated with similar increased all-cause mortality (5). The limitations of the conventional 2-group LVH classification represent possible explanations for this observation. Indeed, since the 2-group LVH classification uses a ratio between the LV cavity diameter and the LV wall thickness, the variations in end-diastolic volume (EDV) and thickness occurring in numerous remodeling patterns cannot be differentiated. Of note, it is known that the simple LV enlargement assessed by echocardiography conveyed independent prognostic utility beyond LV mass for incident heart failure (6).

**Figure 1:**
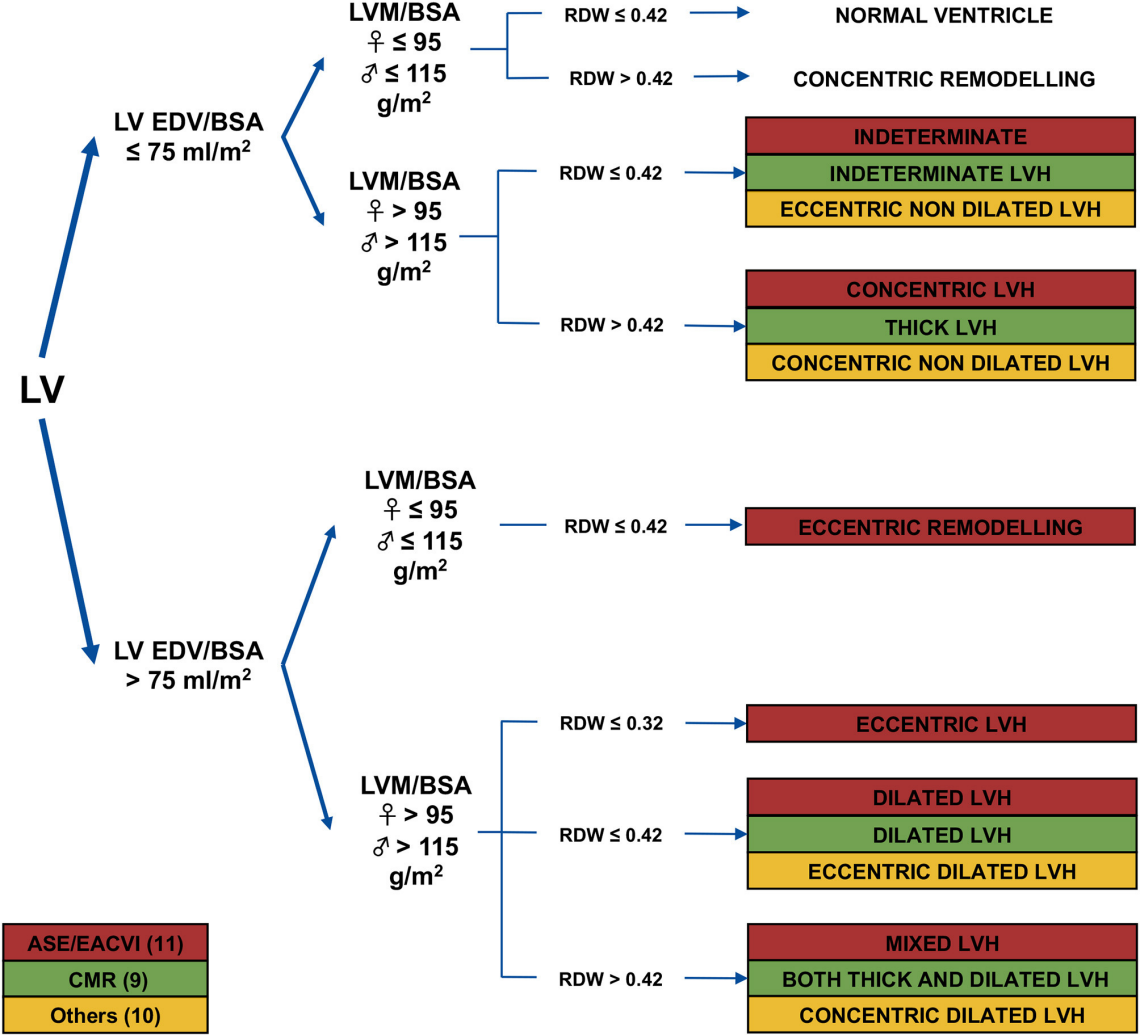
Schematic description of the 4-group left ventricular hypertrophy classification. The different terminology used in the literature and the normal range of parameters under consideration are highlighted. LV, left ventricular; EDV, end-diastolic volume; BSA, body surface area; LVH, left ventricular hypertrophy; RWT, relative wall thickness; ESC, European association of cardiology; CMR, Cardiac magnetic Resonance. Adapted from (8).

**Figure 2:**
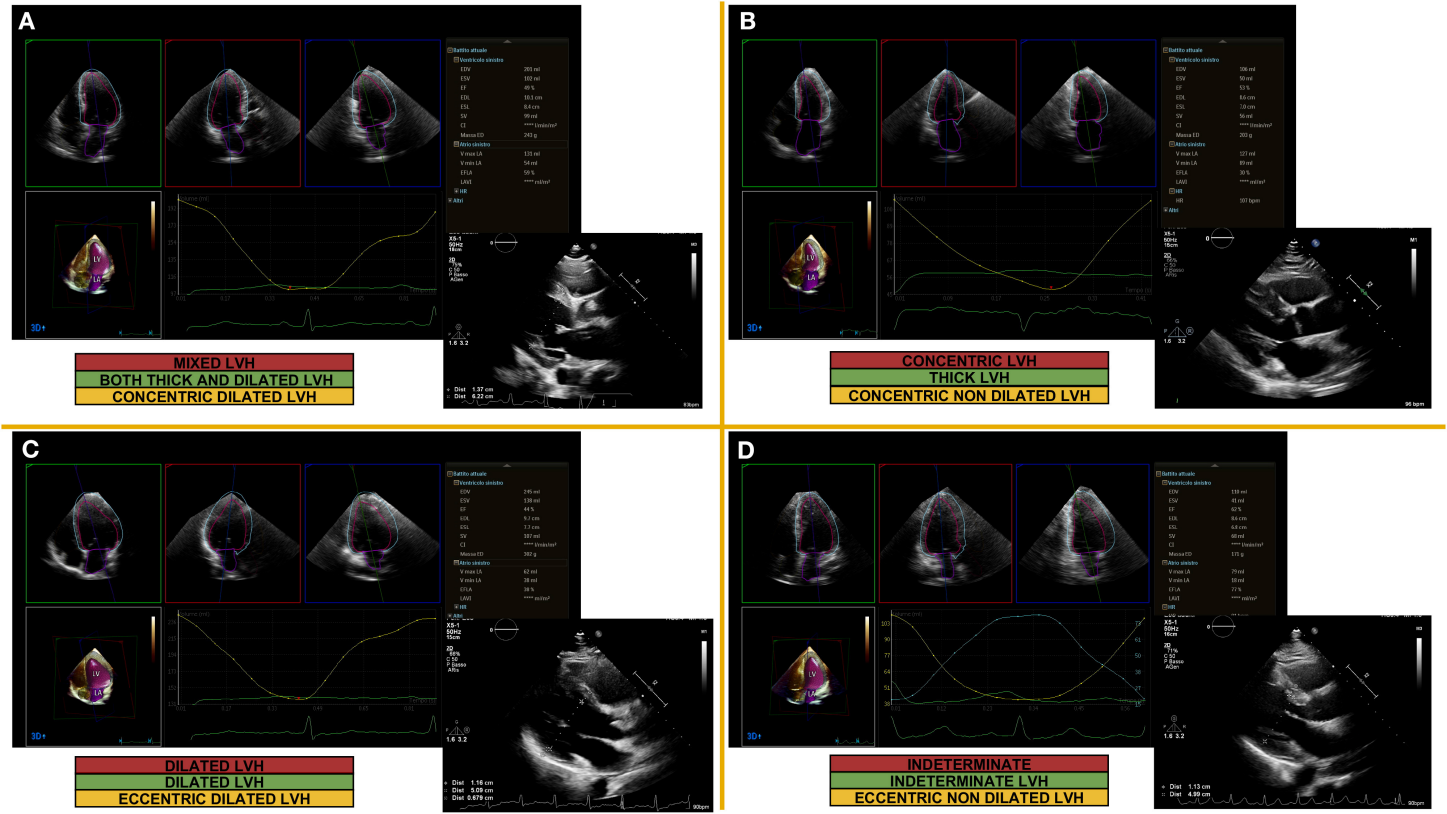
Examples of echocardiographic left lentricular hypertrophy classification based on concentricity, mass and volume quantification. Parasternal long axis view for linear 2D measurements (LV mass, concentricity) and 3D measurements (LV volumes) obtained from automated DHM (Dynamic Heart Model, Philips Healthcare, Andover, MA, USA) in a patient with mixed LVH **(A)**, concentric LVH **(B)**, dilated LVH **(C)**, indeterminate LVH **(D)**; 2D, two-dimensional; 3D, three-dimensional; LV, left ventricular; LVH, left ventricular hypertrophy.

These findings as a whole raise the question of whether there may be other methods to distinguish high-risk phenotypes of LVH. In this context, Gaasch and Zile proposed a subdivision of LVH based on LV mass, EDV and RWT (7). Using this method, a 4-group LVH category based on ventricular concentricity and dilation can be recognized. Depending on EDV dilatation, this classification subdivides both eccentric LVH and concentric LVH into two sub-groups (Figures 1, 2). Essentially, this revised classification assumes that only if the increased LV mass is associated with increased wall thickness and/or ventricular dilation LVH should be considered pathologic.

Although cardiac magnetic resonance (CMR) showed better performance than echocardiography for accuracy and precision in LV mass and volumes assessment could not be served as a routine method for risk assessment of patients with LVH, since it is time-consuming and costly. Accordingly, the purpose of this review is to address the implementation in practice of the novel 4-group LVH classification, focusing on the clinical utility of currently established and widely available echocardiographic techniques.

### Standardization in the Definition of LVH Remodeling Patterns

A definition of the terminology and the normality range of parameters under consideration is required in any LV quantitative analysis. The initially proposed 4-group classification was based on CMR (9). The LVH groups were referred to as: “indeterminate LVH” (neither increased EDV nor concentricity, while LV mass was increased), “dilated LVH” (increased EDV with normal concentricity), “thick LVH” (increased concentricity with normal EDV), and “both thick and dilated LVH” (increased EDV and concentricity). Others have extrapolated successively this classification to echocardiography and have named distinctly the four LVH patterns (e.g., indeterminate LVH as “eccentric non-dilated”; dilated LVH as “eccentric dilated”; thick LVH as “concentric non-dilated”; and both thick and dilated LVH as “concentric dilated”) (10).

More recently, to define the range of normal RWT (0.32–0.42), the ASE/EACVI further divided patients with LVH and EDV dilation into three subgroups: mixed LVH (RWT>0.42), dilated LVH (RWT 0.32–0.42), eccentric LVH (RWT <0.32). Therefore, it was proposed to evolve from the 4-group classification into a new 5-group category to identify subjects with physiological LVH or dilated LVH (e.g., pregnant women, athlete's heart) (11).

We opted to use the ASE/EACVI echocardiographic terminology in the following section for consistency and clarity of this review. Studies that divided patients with LVH and EDV dilation into three subgroups based on RWT are be specifically reported.

### The 4-Group LVH Classification System: Epidemiology

Using the indexation methods and partition values currently proposed by the ASE/EACVI (11), LVH was seen frequently in the general echocardiographic population (42% of subjects). The most common pattern resulted in concentric LVH (16%) (12). However, the LVH pattern's prevalence depended on the population studied (Table 1). Indeed, in patients with isolated severe aortic stenosis, the most frequent remodeling pattern was concentric LVH (57.3%), followed by mixed (18.9%) and dilated LVH (8.4%). Still, the prevalence of the remodeling patterns differed between the symptomatic and asymptomatic patients (20).

**Table 1:** Summary of echocardiographic studies assessing the association of the 4-group left ventricular hypertrophy classification with cardiovascular outcomes.

**References**	**Design**	**Population**	***N***	**Mean FU duration (years)**	**Primary outcome**	**Findings**		
						**LVH group**	**Frequency (%)**	HR (95% CI)^*^
Bang et al. (13)	Prospective	Hypertensive patients	939	4.8	All-cause mortality	No LVH	70	Ref
						Indeterminate	16	NS
						Dilated	1	7.3 (2.8-19)
						Concentric	12	2.4 (1.4-4.0)
						Mixed	0.2	5.8 (1.7-20)
De Simone et al. (14)	Prospective	Hypertensive patients without prevalent CV disease	8,848	2.9	CV mortality, MI, or stroke	No LVH	66	Ref
						Indeterminate	20	NS
						Dilated	3.7	2.0 (1.2-3.1)
						Concentric	5.1	2.2 (1.2-3.8)
						Mixed	0.15	8.9 (2.2-37)
Cuspidi et al. (15)	Prospective	General population without prevalent CV disease	1,694	17.6	All-cause mortality	No LVH	85	Ref
						Indeterminate	6.3	1.6 (1.1-2.3)
						Dilated	3.5	1.9 (1.4-3.4)
						Concentric	4.6	2.2 (1.4-3.4)
						Mixed	0	NA
Barbieri et al. (16)^**^	Retrospective	Aortic valve stenosis (AVA ≤1.5 cm^2^)	343	2.2	All-cause mortality, cardiac hospitalization, or AVR	No LVH	6.9	Ref
						Indeterminate	5.5	NS
						Dilated	3.2	3.7 (1.6-8.5)
						Concentric	39.3	2.6 (1.0-4.7)
						Mixed	22.4	2.6 (1.2-5.8)
Barbieri et al. (12)^**^	Retrospective	Moderate or severe aortic regurgitation	370	3.4	CV mortality, cardiac hospitalization, or AVR	No LVH	26.2	Ref
						Indeterminate	12.2	NS
						Dilated	14.6	7.9 (1.8-34.3)
						Concentric	11.6	NS
						Mixed	10.3	4.3 (1.0-19.9)
Huang et al. (17)	Prospective	Coronary artery disease	2,297	2.1	All-cause mortality	No LVH	60	Ref
						Indeterminate	6	NS
						Dilated	10	2.8 (1.7-4.3)
						Concentric	19	1.7 (1.1-2.6)
						Mixed	5	2.3 (1.3-4.1)
Pugliese et al. (18)	Prospective	Asymptomatic heart failure (stage A and B)	1,729	1.7	All-cause mortality, myocardial infarction, coronary revascularizations, cerebrovascular events, and acute pulmonary edema	No LVH	70.1	Ref
						Indeterminate	NA	NA
						Dilated	2.7	3.1 (1.5-3.5)
						Concentric	18	1.9 (1.1-3.1)
						Mixed	1.5	2.3 (1.3-4.1)
Wang et al. (19)	Cross-sectional	General population of China	11,037	NA	Non-fatal ischemic stroke	No LVH	88.6	Ref
						Indeterminate	4.3	1.6 (1.1-2.3)
						Dilated	3.4	NS
						Concentric	2.2	2.1 (1.3-3.4)
						Mixed	1.2	NS

*CI, confidence interval; HR, hazard ratio; NA, not applicable; NS, not significant; Ref, referent group*.

* *Hazard ratios are reported with the 95% confidence interval. Participants without hypertrophy are the referent group*.

** *In this study patients with LVH and EDV dilation were divided into three subgroups: mixed LVH (RWT> 0.42), dilated LVH (RWT 0.32–0.42), eccentric LVH (RWT <0.32)*.

### The 4-Group LVH Classification System: Association With Hemodynamic Profiles and Biological Markers

It is known that the curvilinear inverse relation between EF and EDV generally predicts that EF would be depressed when the LV is dilated and preserved when the volume is normal (21). The LV pump function's normality depends on maintaining the double-helical (spiral) alignment of the LV myocardial architecture determined by the LV geometry. In concentric LVH with normal EDV, the LV myocardial architecture's double-helical orientation is preserved, resulting in a normal or near-normal EF. Conversely, the LV myocardial architecture's double-helical orientation is disrupted in the eccentric LVH with unbalanced EDV dilatation, resulting in decreased EF (22). Therefore to be useful in clinical practice, the LVH remodeling patterns must be accompanied by compatible hemodynamic and functional profiles plausible from a pathophysiological perspective.

In the original Dallas heart study, the four geometric patterns of LVH were associated with different clinical characteristics, biomarkers, and ejection fractions. Compared with subjects with concentric LVH, those with mixed LVH had a lower EF and higher NT-pro-BNP and BNP levels (*P* < 0.001 for all). Subjects with dilated LVH had a lower EF and higher troponin T, NT-pro-BNP, and BNP levels versus those with indeterminate LVH (*P* < 0.001 for all). Subjects with indeterminate LVH had no elevation of markers of cardiac stress as compared with subjects without LVH (9).

These findings were extended to a sizeable echocardiographic population focusing on applying contemporary proposed indexation methods and partition values. The worst hemodynamic profile was associated with eccentric LVH. The prevalence of diastolic dysfunction (defined as mean E/E′ > 14) was 43.5% in subjects with eccentric LVH, 36% in those with dilated LVH, 20.7% in concentric LVH and 8.2% in patients without LVH (*P* < 0.0001). The prevalence of pulmonary hypertension (defined as derived pulmonary artery pressure ≥ 50 mmHg) was 25.7% in subjects with eccentric LVH and 1.9% in those without LVH (*P* < 0.0001) (12).

Similarly, The 4-group classification was correlated with LV mechanics in a cohort of hypertensive patients. Those with concentric, dilated, and mixed LVH had longitudinal, circumferential, and radial strain unfavorably affected after adjusted analysis. Of note, there was no substantial difference in strain for those with indeterminate LVH and those without LVH (15).

The new 4-group classification also showed higher discrimination of exercise-induced LVH patterns in a cohort of normotensive endurance athletes relative to the existing 2-group classification (8). Besides, other studies tested the association of the 4-group classification with biological markers. In hypertensive patients, dilated or mixed LVH was associated with an increased prevalence of subclinical renal damage (23), patients with metabolic syndrome had a higher prevalence of dilated or mixed LVH (24). Therefore, it appears that the proposed new LVH subcategories are not only mere descriptors of LV geometry but an integral component of parameters reflecting systolic properties.

### The 4-Group LVH Classification System and Clinical Outcomes

To resolve the dynamic relationship between LV dilation and myocardial thickening in LVH pathophysiology, several echocardiographic studies have linked the 4-group classification system to clinical outcome in hypertensive patients (13, 14), patients with coronary artery disease (17), patients with asymptomatic (stage A and B) heart failure (18), in the general population with normal LV systolic function and no history of heart failure (25), and patients with valvular heart disease (16, 26).

Compared to participants without LVH, the 4-group LVH classification system was a robust prediction model of adverse cardiovascular outcomes in all these studies Table 1. The Losartan Intervention for Endpoint Reduction Echocardiography sub-study was the first to use readily available echocardiographic measurements to reproduce the results of CMR (9) in 939 hypertensive patients who were treated for 4.8 years. They found that of all-cause mortality risk was increased for patients with dilated, concentric, and mixed LVH [HR (95%CI)]: 7.3 (2.8–19), 2.4 (1.4–4.0), 2.4 (1.4–4.0), respectively. The same result was found for cardiovascular mortality and the composite endpoint of myocardial infarction, stroke, heart failure, and cardiovascular mortality. On the other hand, indeterminate LVH was not associated with increased relative risk compared to patients without LVH identifying a low-risk group with eccentric LVH and the same risk of all-cause mortality or cardiovascular events such as patients with normal LV mass (13). Nevertheless, in this study some of the LVH subgroups had a limited number of endpoints, thus only the primary endpoint of all-cause mortality was adjusted for multiple comparisons, reducing the power to verify the incremental prognostic value of the 4-group system in the two concentric LVH groups.

The largest cohort of 8,848 hypertensive patients with no history of cardiovascular disease from the Campania Salute Network paralleled these findings, showing that patients with indeterminate LVH were not at increased risk compared to those without LVH. Conversely, there was a substantial increase in the incidence of fatal and non-fatal cardiovascular and cerebrovascular accidents in patients with dilated, concentric, and mixed LVH compared to those without LVH [HR (95%CI)]: 2.0 (1.2–3.1), 2.2 (1.2–3.8), 8.9 (2.2–37), respectively (14). This was the first direct evidence that differences in left ventricular geometry may be relevant to the definition of risk profile in a large community-based registry of uncomplicated hypertensive patients.

Huang et al. applied the 4-group LVH classification to 2,297 patients with angiographic evidence of stable coronary artery disease and reported outcomes after a 2-year follow-up. Patients with dilated, concentric, and mixed LVH were at increased risk of all-cause mortality compared with those without LVH [HR (95%CI)]: 2.8 (1.7–4.3), 1.7 (1.1–2.6), 2.3 (1.3–4.1), respectively. Once more, the risk of primary or secondary endpoints was not increased in participants with indeterminate LVH (17). However, only baseline echocardiography data were available, and the modification in LV geometry during interventional and medical therapy was unknown. This is an important limitation considering that in hypertensive patients, only “in-treatment” LV geometry by echocardiography predicted risk of cardiovascular events, but not baseline LV geometry (27).

Our group evaluated the application of the novel LVH classification in patients with valvular heart disease. In 342 patients with aortic stenosis (functional aortic valve area ≤1.5 cm^2^), there was a significant association between adverse events and LV dilatation or LV remodeling pattern. After multivariate adjustment, dilated, concentric, and mixed LVH were strongly associated with death or cardiac hospitalization [HR (95%CI)]: 3.7 (1.6–8.5), 2.6 (1.0–4.7), 2.6 (1.2–5.8), respectively (16). In 370 consecutive patients with moderate or severe chronic aortic regurgitation, dilated and mixed LVH were associated with the combination of cardiovascular death, hospitalization for acute heart failure, or aortic valve replacement [HR (95%CI)]: 7.9 (1.8–34.3), 4.3 (1.0–19.9), respectively (26). In the Pressioni Monitorate e Loro Associazioni (PAMELA) study, dilated and concentric LVH predicted cardiovascular and all-cause mortality risk in the general population without valve disease and with normal EF after an average follow-up of 17.5 years [HR (95%CI)]: 1.9 (1.4–3.4), 2.2 (1.4–3.4), respectively. In contrast to the above studies, even indeterminate LVH demonstrated independent prognostic value [HR (95%CI)]: 1.6 (1.1–2.3). This may be due to the longer follow-up than other studies. However, the threshold criterion to define increased RWT was 0.45 and 0.44 for men and women, respectively, which was slightly higher than the ASE/EACVI guideline cutoff of 0.43. Of note, only concentric LVH maintained a significant predictive value for both outcomes after adjusting for baseline differences in the LV mass index (25).

In a multicenter study designed by the Italian Society of Echography and Cardiovascular Imaging (SIECVI), the novel 4-group classification was an independent predictor of adverse events during follow-up in 1.750 patients with stage A or B heart failure. Remarkably, it produced a better risk stratification in comparison to the classic 2-group one. The worst prognosis was reported for patients with dilated, concentric, and mixed LVH compared to those without LVH [HR (95%CI)]: 3.1 (1.5–3.5), 1.9 (1.1–3.1), 2.3 (1.3–4.1), respectively (18). The primary study limitation was the use of composite outcomes (all-cause death, myocardial infarction, coronary revascularization, cerebrovascular event, and acute pulmonary edema) due to the low prevalence of adverse events for the study population size and follow-up length.

In a large population from China with low cardiovascular risk, the presence of concentric and indeterminate LVH was associated with an increased risk of non-fatal ischemic stroke [HR (95%CI)]: 2.1 (1.3–3.4), 1.6 (1.1–2.3), respectively. Surprisingly, dilated and mixed LVH were not associated with an increased risk of ischemic stroke (19). Surprisingly, dilated and mixed LVH were not associated with an increased risk of ischemic stroke. Due to the cross-sectional nature of this study, it was difficult to assign causality to these findings. One explanation may be partially attributed to worse cardiometabolic risk factors for individuals with indeterminate LVH. However, in multivariable analysis, LV concentricity, but not LVEDV, was a significant predictor of ischemic stroke when analyzed as continuous variables. It is possible to hypothesize that both the heart and the brain are potential organs at risk for injury as a result of long-term elevated blood pressure, which emerges as critical correlations between LVH and ischemic stroke (28, 29). Furthermore, after adjusting for conventionally measured blood pressure, carotid disease was considered to parallel LV mass (30, 31) and represented a particularly sensitive marker of ischemic stroke (32).

Overall, these observational data suggest that the incorporation of LV chamber dilation into the assessment of LVH identifies important sub-phenotypes within the standard 2-group classification. In particular, LVH with RWT ≤0.42 can be split into a low-risk group (indeterminate LVH) and a high-risk group (dilated LVH). Similarly, the two phenotypes of LV dilatation (dilated and mixed LVH) should be considered a high-risk LVH phenotype.

### The 4-Group LVH Classification System: A Critical Viewpoint

It is necessary to recognize some potential limitations of the new 4-group LVH classification for its correct use in clinical practice. The 4-group LVH classification scheme is necessarily definite and based on numerical thresholds to be usable. However, for some authors this dichotomous definition of LVH should be reconsidered and analyzed as a continuum from normal to remodeling, with possible implications for reverse remodeling (10). Recently, Yamanaka et al., using a landmark analysis in patients with clinical heart failure and an EF ≥ 50%, found that, compared with patients without LVH or LV enlargement at baseline, subsequent adverse outcomes were more frequent in patients with LVH without LV enlargement at baseline and were even more frequent in patients with LV enlargement (33). In addition to examining the predictive value of LV mass and LVEDV as categorical variables, the authors also examined them as continuous variables: the relationships between structural category and outcome remained unchanged within a multivariate analysis adjusting for clinical variables including EF.

Also, a patient can move between categories only based on limitations in the reproducibility of echocardiographic measurements (34). At the same time, it may reflect a transitional pattern of dynamic temporal change. Previous data suggested that changes in LV geometry over time may impact prognosis similarly to many studies suggesting improvements in prognosis with regression of LVH (35). Therefore, the trajectory of the LVH pattern more than a single evaluation may be necessary to further classify patients with LVH.

The consideration of EDV, mass, and RWT does not allow all LVH remodeling changes to be classified (12% of consecutive patients for any echocardiographic indication) (12). However, in cardiology, this gap in classification is not new. Whether this misclassification has clinical implications should be investigated in more extensive research (36). Some studies are limited because they combined patients with dilated and eccentric LVH into the same group (dilated LVH). Still, these two groups may probably have a differential risk of developing incident heart failure. The dilated LVH pattern could not be differentiated by “physiological LVH,” such as athlete's heart with an early stage of a pathological condition. However, by contextualizing the clinical environment and the degree of LV dilation, these LVH profiles are easily detectable.

It should be stressed that the determination of RWT can be limited by non-uniform wall thickness and regional shape deformation (37). Three-dimensional echocardiography (3D), without geometric assumptions about LV form and wall thickening distribution, is the only echocardiographic technique that accurately measures the LV mass in these patients (38). It is worth noting that in hypertensive patients, a high 3D-LV mass/EDV ratio identified a higher incidence of concentric LVH compared to 2D-derived relative wall thickness, which is inversely correlated with the stroke volume (39) and early systolic and diastolic dysfunction (40).

The current normative values are derived from 2D-echocardiography. With the advent of artificial intelligence and automated 3D approach to LV chamber quantification (Figure 2), echocardiographic quantification practice will be changed soon and new and gender-specific cut-off values will be proposed (41).

While CMR outperformed echocardiography in terms of accuracy and precision in LV mass evaluation, no clear comparison of the two methods has been done for the ability to predict clinical events, LVH classification agreement, or cardiovascular risk reclassification.

Other LVH diagnostic methods have their risk profile, independent and complementary to the LV mass detected by echocardiography. A previous analysis from the Cardiovascular Health Study showed that both LVH detected by electrocardiography and echocardiography was predictive of future atrial fibrillation events, independent of well-known risk factors, suggesting that LVH detected by electrocardiography is an important electrophysiological marker of cardiac abnormalities independent of LV mass detected by echocardiography (42).

Finally, because the vast majority of subjects enrolled in studies were Caucasian, results may not apply to other ethnicities (43).

## Perspectives

A key finding of the improved LVH phenotypic characterization is the demonstration of a wide range of changes in EDV, mass, and function in patients with chronic heart failure. Therefore, the main questions are whether some of the differences in LV remodeling response are due to differences in the lesion or discrepancies in the host's lesion response. However, the question will be almost impossible to address in clinical practice (44). Indeed, the occurrence of LVH geometric anomalies showed considerable variability in patients with the same heart disease (13, 17, 35, 37, 45–50). Several factors, including but not limited to gender, diabetes, previous myocardial infarction, obesity, and valve diseases, tended to affect the remodeling of LVH (51). Besides, there are likely underlying genetic factors that remain poorly identified (52). Therefore, refining the classification of LVH could distinguish the distinctive development of LV geometric changes from baseline and the transition to a maladaptive phase of remodeling in the individual patient (10). Using the conventional 2-group categorization, previous echocardiographic longitudinal studies showed that progression from concentric LVH to eccentric LVH occurred in 19% of subjects after 4 years (53) and 25% after 7 years (54). Nevertheless, how often those who converted to eccentric LVH had a dilated LV at follow-up was not mentioned. Recent CMR data indicated that in hypertensive patients, concentric LVH dilated less often than previously assumed over an extended timeframe in the absence of interval myocardial infarction (55).

Enhanced LVH characterization will also provide opportunities for LV geometry-directed therapeutic intervention in order to reduce incident heart failure. Recent data suggested that, in patients with heart failure with reduced EF, patients with concentric LVH did not experienced similar benefits from up-titration angiotensin-converting enzyme inhibitors/angiotensin receptor blockers and beta-blockers compared to patients with eccentric LVH (4).

## Conclusions

Chronic heart failure is a dynamic clinical condition with a broad phenotypic variability that makes the “one-size-fits-all” approach inadequate to care. In clinical practice, many patients have a combination of chronic pressure and volume overload, leading to distinct and more complex LVH geometric patterns than previously considered, underscoring the need for a better LVH classification. With this in mind, conventional 2-group patterns are not adequate for risk stratifying patients with LVH. Conversely, preliminary findings supported the use of LV remodeling assessment based on EDV, mass, and RWT by echocardiography. As clinicians, it is time to start thinking about new LVH classification proposals that will consider many parameters of LV morphology and function, including underlying remodeling abnormalities that can be obtained with current echocardiographic technology. However, further evidence is needed to understand how it can be integrated into clinical decision-making.

## Author Contributions

AB: substantial contributions to the conception or design of the work and agree to be accountable for all aspects of the work in ensuring that questions related to the accuracy or integrity of any part of the work are appropriately investigated and resolved. AB, AA, AM, GD, GC, and NB: acquisition, analysis or interpretation of data for the work. AB, GB, AA, AM, FM, GD, GC, and NB: drafting the work or revising it critically for important intellectual content and provide approval for publication of the content. All authors contributed to the article and approved the submitted version.

## Conflict of Interest

The authors declare that the research was conducted in the absence of any commercial or financial relationships that could be construed as a potential conflict of interest.
